# Plasmalogen Augmentation Reverses Striatal Dopamine Loss in MPTP Mice

**DOI:** 10.1371/journal.pone.0151020

**Published:** 2016-03-09

**Authors:** Edith Miville-Godbout, Mélanie Bourque, Marc Morissette, Sara Al-Sweidi, Tara Smith, Asuka Mochizuki, Vijitha Senanayake, Dushmanthi Jayasinghe, Li Wang, Dayan Goodenowe, Thérèse Di Paolo

**Affiliations:** 1 Neuroscience Research Unit, Centre de Recherche du CHU de Québec, CHUL, Quebec City, Canada; 2 Faculty of Pharmacy, Laval University, Quebec City, Canada; 3 Phenomenome Discoveries Inc., Saskatoon, Canada; 4 Department of Molecular Diagnosis, Graduate School of Medicine, Chiba University, Chiba, Japan; Hudson Institute, AUSTRALIA

## Abstract

Plasmalogens are a class of glycerophospholipids shown to play critical roles in membrane structure and function. Decreased plasmalogens are reported in the brain and blood of Parkinson’s disease (PD) patients. The present study investigated the hypothesis that augmenting plasmalogens could protect striatal dopamine neurons that degenerate in response to 1-methyl-4-phenyl-1,2,3,6-tetrahydropyridine (MPTP) treatment in mice, a PD model. First, in a pre-treatment experiment male mice were treated for 10 days with the docosahexaenoic acid (DHA)-plasmalogen precursor PPI-1011 (10, 50 and 200 mg/kg). On day 5 mice received MPTP and were killed on day 11. Next, in a post-treatment study, male mice were treated with MPTP and then received daily for 5 days PPI-1011 (5, 10 and 50 mg/kg). MPTP treatment reduced serum plasmalogen levels, striatal contents of dopamine (DA) and its metabolites, serotonin, DA transporter (DAT) and vesicular monoamine transporter 2 (VMAT2). Pre-treatment with PPI-1011 (10 and 50 mg/kg) prevented all MPTP-induced effects. Positive correlations were measured between striatal DA contents and serum plasmalogen levels as well as striatal DAT and VMAT2 specific binding. Post-treatment with PPI-1011 prevented all MPTP-induced effects at 50 mg/kg but not at lower doses. Positive correlations were measured between striatal DA contents and serum plasmalogen levels as well as striatal DAT and VMAT2 specific binding in the post-treatment experiment. PPI-1011 treatment (10 days at 5, 10 and 50 mg/kg) of intact mice left unchanged striatal biogenic amine contents. These data demonstrate that treatment with a plasmalogen precursor is capable of protecting striatal dopamine markers in an animal model of PD.

## Introduction

The cause of the majority of Parkinson’s disease (PD) cases is currently unknown and there is presently no cure for PD [1]. Neuroprotective or disease modifying interventions capable of protecting or rescuing vulnerable neurons, thereby slowing, stopping, or reversing disease progression is not yet available for PD.

Ethanolamine plasmalogens (PlsEtns) are a unique class of glycerophospholipids, which play a critical role in membrane structure mediated functions such as vesicular release of neurotransmitters, membrane protein activity and free radical scavenging. They also act as a storage depot of neuroprotective polyunsaturated fatty acids [2] such as docosahexaenoic acid (DHA) [3]. PlsEtns constitute about 30 mol% of the total brain phospholipids [4], and about 70% of all glycerophospholipids in myelin [5]. Critical steps in plasmalogen biosynthesis take place in peroxisomes. Peroxisomal biogenesis disorders like Rhizomelic Chondro Dysplasia Punctata has severe plasmalogen deficiency accompanied by serious neurological deficits highlighting the significance of plasmalogens for neurological function (as reviewed in [2]). Rationale for this study comes from the observations of reductions in PlsEtn concentrations in serum of patients with PD [6] and in the frontal cortex lipid rafts of PD patients [7].

**Fig 1:**
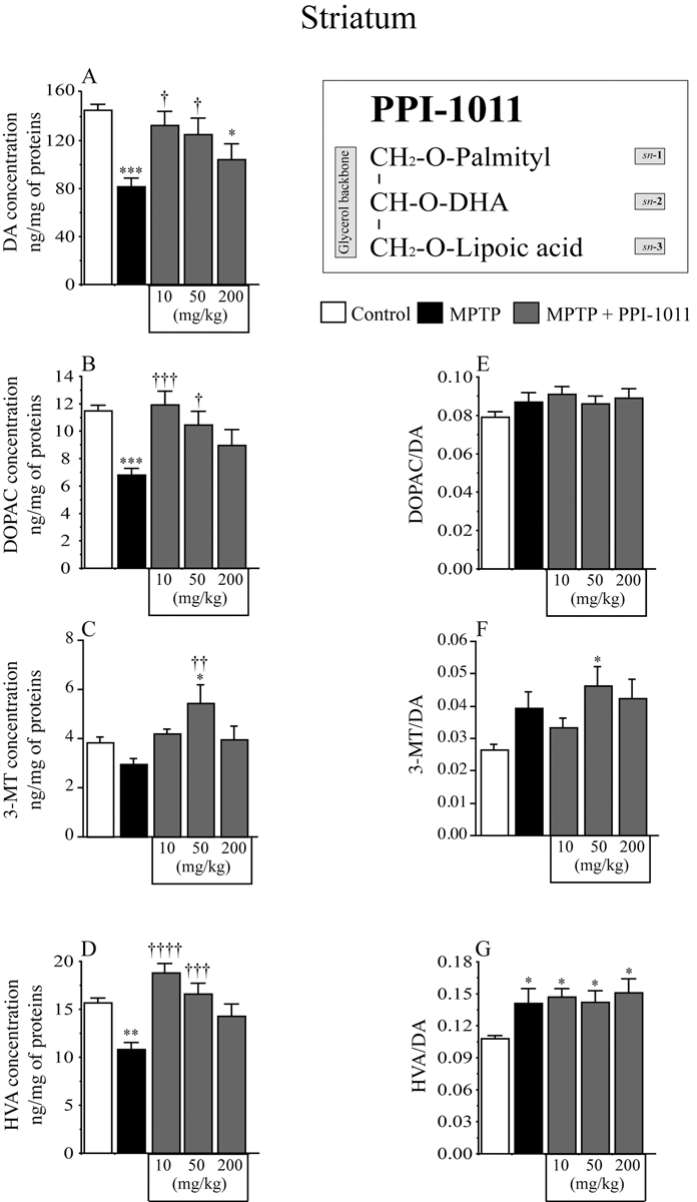
PPI-1011 pre-treatment neuroprotection of striatal dopamine and its metabolites. Effect of MPTP and PPI-1011 treatment on striatal A) dopamine (DA) contents (*F*_4,46_ = 5.91, *p* = 0.0006) and its metabolites B) 3,4-dihydroxyphenylacetic acid (DOPAC) (*F*_4,46_ = 6.17, *p* = 0.0005), C) 3-methoxytyramine (3-MT) (*F*_4,46_ = 3.90, *p* = 0.008), and D) homovanillic acid (HVA) (*F*_4,46_ = 9.32, *p* < 0.0001), as well as E) DOPAC/DA (*F*_4,46_ = 1.16, *p* = 0.34), and F) 3-MT/DA (*F*_4,46_ = 3.03, *p* = 0.03) and G) HVA/DA (*F*_4,46_ = 3.08, *p* = 0.02) ratios. Values shown are the means (ng/mg of proteins) ± S.E.M. of 9–12 mice per group. * p < 0.05, ** p < 0.01 and *** p < 0.001 vs control; † p < 0.05, †† p < 0.01, ††† p < 0.001 and †††† p < 0.0001 vs MPTP.

PPI-1011 (Fig 1) is an orally bioavailable PlsEtn precursor that bypass peroxisomal steps of plasmalogen synthesis to generate PlsEtn species that can eventually reach the brain [8]. It can also acts as a sustained release preparation of the sn-2 fatty acid DHA and the sn-3 lipoic acid. Experiments in rabbits have demonstrated that PPI-1011 increased serum steady-state levels of DHA and DHA-PlsEtn [9]. DHA is known to be neuroprotective [3]. Since lipoic acid enhances glutathione levels, its protective effect against oxidative stress associated with dopaminergic neuronal cell death could offer potential neuroprotective effects as well [10, 11].

The 1-methyl-4-phenyl-1,2,3,6-tetrahydropyridine (MPTP) neurotoxic model of PD is widely used to test drugs for neuroprotective activity [12]. This model originates from discoveries of drug users that showed severe symptoms similar to PD [13, 14]; their investigation showed that they self-administered a synthetic meperidine contaminated with MPTP [14]. Since its discovery, MPTP toxicity has been extensively investigated [15]. The MPTP mouse shows loss of substantia nigra dopamine (DA) neurons and striatal DA content depletion as observed in PD [16]. Moreover, men with PD are reported to have lower testosterone blood levels that are also modeled in MPTP male mice [17, 18]. In addition, PD is associated with gastrointestinal dysfunctions and we have shown changes of various markers in the myenteric plexus of MPTP mice [19]. Therefore, the MPTP mouse offers a good and rapid *in vivo* model to test neuroprotective drugs.

The present study first investigated the hypothesis that PlsEtn are reduced in the MPTP model of PD. Since reductions of PlsEtn were observed in the MPTP model, similar to previously cited PlsEtn reductions in patients, we then investigated PlsEtn augmentation with an orally bioavailable PlsEtn precursor as a potential preventive/restorative mechanism. Treatments with PPI-1011 both pre- and post MPTP exposure, were evaluated for preventive and restorative effects respectively. Dopaminergic system was investigated as the efficacy parameter of preventive/restorative effects of PPI-1011.

## Methods

### Animals and Treatment

C57Bl/6 male mice (10 weeks) were purchased from Charles River Canada (Montreal, QC, Canada). PPI-1011 was provided by Phenomenome Discoveries Inc. (Saskatoon, SK, Canada). The mice were handled in accordance with the National Institute of Health Guide for the Care and Use of Laboratory Animals. The Laval University Animal Care Committee approved this study. All efforts were made to minimize animal suffering and to reduce the number of mice used.

### PPI-1011 pre-MPTP treatment experiment

Each group (9–12 animals per group, 51 in total) received treatment with vehicle (0.1 ml soybean oil, administered orally once daily) or PPI-1011 (10, 50 and 200 mg/kg, formulated in 0.1 ml soybean oil, administered orally once daily) for 10 days. On day 5, mice received four injections of MPTP (6.5 mg/kg i.p., Sigma Chemical, St. Louis, MO) at 2-h intervals, whereas the control group received saline solution. A high and extensive range of PPI-1011 dose was chosen for this first dose finding experiment. The dose regimen of MPTP was chosen to obtain a moderate loss of striatal DA contents, representative of an early stage of PD. Under these moderate lesion conditions substantia nigra DA neurons are spared while their terminals in the striatum are degenerated [20–22]. On day 11, mice were decapitated, trunk blood was collected, and brains were quickly removed and frozen in isopentane (-40°C).

### PPI-1011 post-MPTP treatment experiment

Each group (9–14 animals per group, 54 in total) received treatment with vehicle (0.1 ml soybean oil, administered orally once daily) or PPI-1011 (5, 10 and 50 mg/kg, formulated in soybean oil 0.1 ml, administered orally once daily) for 5 days starting the day of MPTP administration which consisted of four injections of MPTP (6.5 mg/kg i.p., Sigma Chemical, St. Louis, MO) at 2-h intervals, whereas the control group received saline solution. The PPI-1011 doses chosen were lower in this next experiment and based on the pre-treatment experiment. The day of MPTP injections one PPI-1011 oral administration was given 1 h after the first MPTP injection and another 1 h after the last MPTP injection. For the following days PPI-1011 was administered once daily in the morning. On day 6, mice were decapitated, trunk blood was collected, and brains were quickly removed and frozen in isopentane (-40°C).

### PPI-1011 experiment in intact mice

Each group (10 animals per group, 40 in total) received treatment with vehicle (0.1 ml soybean oil, administered orally once daily) or PPI-1011 (5, 10 and 50 mg/kg, formulated in 0.1 ml soybean oil, administered orally once daily) for 10 days. On day 11, mice were decapitated, trunk blood was collected, and brains were quickly removed and frozen in isopentane (-40°C).

### Brain preparation

The striatum was dissected from one half brain to assay biogenic amine and their metabolites contents. From this same half brain the posterior striatum (bregma 0.50 to -0.70 mm) and the substantia nigra (bregma -2.92 at -3.64 mm) [23] were cut on a cryostat in 12 μm slices for autoradiography. The other half brain and the cerebellum were pooled and used for plasmalogen concentration measures. Brain samples were kept at– 80°C until assayed.

### Striatal biogenic amine assay

The left anterior striata were dissected, homogenized in 250 μl of 0.1N HClO_4_ at 4°C and then centrifuged at 10 000g for 10 min (4°C) to precipitate proteins. The contents of DA and its metabolites 3,4-dihydroxyphenylacetic acid (DOPAC), 3-methoxytyramine (3-MT) and homovanillic acid (HVA) as well as serotonin and its metabolite 5- hydroxyindoleacetic acid (5-HIAA) were measured by high performance liquid chromatography (HPLC) with electrochemical detection as previously described with slight modifications [24]. Supernatants of striatal tissue were directly injected into the chromatograph consisting of a Waters 717 plus autosampler automatic injector, a Waters 515 pump equipped with a C-18 column (Waters Nova-Pak C18, 3 μm, 3.9 mm x 150 cm), a BAS LC-4C electrochemical detector and a glassy carbon electrode. The mobile phase consisted of 0.025 M citric acid, 1.7 mM 1-heptane-sulfonic acid, and 10% methanol, in filtered distilled water, delivered at a flow rate of 0.8 ml/min. The final pH of 4.1 was obtained by addition of NaOH. The electrochemical potential was set at 0.8 V with respect to an Ag/AgCl reference electrode. Results were expressed in nanograms of amine per milligram of protein. Proteins were assayed with a Micro BCA Protein Assay kit (Thermo Scientific, Rockford, IL).

### Dopamine transporter (DAT) and vesicular monoamine transporter 2 (VMAT2) autoradiography

Autoradiography of striatal DAT specific binding used 20 pmol of the ligand 3ß-(4-[^125^I] iodophenyl) tropane-2ß-carboxylic acid isopropyl ester ([^125^I]-RTI-121) (2200 Ci/mmol; PerkinElmer, Boston, MA, USA). Binding in presence of 100 nM of mazindol (Sandoz, Pharmaceuticals, Dorval, QC, Canada) was used to estimate non-specific binding [25]. Brain slices were exposed 30 h for the striatum and 20 h for the substantia nigra on Kodak BioMax MR films (Eastman Kodak Company, Rochester NY, USA).

VMAT2 autoradiography [26] was performed using the specific ligand [^3^H] dihydrotetrabenazine ([^3^H]-TBZ-OH) (20 Ci/mmol). Binding in presence of 1 μM of cold dihydrotetrabenazine was used to estimate non-specific binding (American Radiolabeled Chemicals, St-Louis, MO, USA). Brain slices of striatum and substantia nigra were exposed 6 weeks on Kodak BioMax MR films (Eastman Kodak Company, Rochester NY, USA).

Analyses of films were made using NIH Image 1.63 software (developed at the USA National Institutes of Health; http://rsb.info.nih.gov/nih-image/). One glass slide per animal (containing 4 to 6 consecutive brain sections) was used for each binding experiment thus the data presented for each animal is the mean of data from 4 to 6 brain sections. Specific binding was calculated by subtracting non-specific binding from total binding.

### Quantification of plasmalogens and metabolites in brain and serum

Blood samples were collected in serum tubes and centrifuged (10 min, 1000g, 4°C). Serum samples were stored at -80°C until thawed for analysis. 10 μl of internal standards (1 μg/ml of ^13^C-PlsEtn (C3713C6H74NO7P) and 1 μg/ml of ^13^C-PtdEtn (C2413C19H74NO8P) in dichloromethane) were evaporated at room temperature and added to 20 μl of serum, 40 μl of 4% formic acid in water and 0.5 ml of 100% ethyl acetate (EtOAc). After vortexing the mixture for 15 min, samples were centrifuged at 4°C for 2 min at 2000g. The EtOAc organic fraction was used for the analysis of each sample. All extracts were stored at -80°C until analysis. High-throughput analysis was performed with a triple quadrupole mass spectrometer (IONICS 3Q, IONICS) coupled with the Agilent 1100 LC system. A 100 μl sample was injected by flow injection analysis and monitored under negative atmospheric pressure chemical ionization (APCI) mode. The method was based on multiple reaction monitoring (MRM) of one parent/fragment transition for the ion pairs. Each transition was scanned for 50 ms. EtOAc: methanol: water: formic acid ratio of 38.5: 70: 1: 0.5 at a flow rate of 600 μl /min was used as the mobile phase. All solvents were HPLC grade. The list of analytes and their MRM transitions for quantification of plasmalogens and their metabolites in serum samples are listed in Table A in S1 File. The total acquisition time per sample was 1 min. Stable isotope ratios for each analyte were calculated.

Brain samples were homogenized using liquid nitrogen, first with a mortar and pestle followed by the TissueLyser LT (Qiagen, Toronto, ON, Canada) generating a fine powder. Tissues were aliquoted with anti-static polypropylene disposable milligram scoops (TWD Tradewinds, Pleasant Prairie, WI, USA). Water (200 μl) was added and samples were ice bath sonicated for 30 min prior to the addition of 600 μl of EtOAc. Samples were stirred at 2000 rpm for 15 min followed by a 10 min centrifugation at 2851g. An aliquot (36 μl) of the upper organic layer was then diluted in a solution of water and EtOAc (420 μl) containing the same 2 labeled internal standards used above. Samples were again stirred at 2000 rpm for 15 min followed by a 2 min centrifugation at 2851g. High-throughput analysis method was based on multiple reaction monitoring (MRM) of one parent/fragment transition for the ion pairs, performed using LC/MS (Agilent 1100 HPLC pump and API4000™ mass spectrometer equipped with a TurboV™ source using NAPCI probe). A 100 μl sample was injected by flow injection analysis and each transition was scanned for 50 ms with a total acquisition time per sample of 1 min. EtOAc: methanol: water: formic acid ratio of 38.5: 70: 1: 0.5 at a flow rate of 600 μl /min was used as the mobile phase. The list of analytes and their MRM transitions for quantification of plasmalogens and their metabolites in brain tissue samples are listed in Table B in S1 File. Stable isotope ratios for each analyte were calculated.

All standards and stable isotopes used in both serum and tissue analyses were >95% pure and manufactured by Phenomenome Discoveries Inc. Solutions used were all HPLC grade.

### Statistical analysis

Statistical analysis of the data were performed with a one-way analysis of variance (ANOVA) using Prism 6 (version 6.0a) for Macintosh Computer software, followed by a post-hoc analysis with a Holm-Sidak’s multiple comparison test. Only statistical comparisons of results to controls or vehicle-treated MPTP mice were performed. A p < 0.05 was required for the results to be considered statistically significant. A simple regression model was used to determine coefficients of correlation and the significance of the degree of linear relationship between the striatal DA markers values and serum PlsEtn concentrations.

## Results

In the pre-treatment PPI-1011 dose finding experiment, MPTP induced a decrease in striatal DA contents which was prevented by PPI-1011 treatment at 10 and 50 mg/kg while the highest dose of 200 mg/kg did not provide protection (Fig 1A). Moreover, PPI-1011 treatment at 10 and 50 mg/kg prevented the MPTP-induced decrease of striatal DOPAC (Fig 1B) and HVA (Fig 1D) contents. The PPI-1011 treatment at 200 mg/kg did not protect against striatal loss of MPTP-induced DOPAC and HVA contents. Striatal 3-MT contents (Fig 1C) were not significantly reduced by MPTP lesion and were increased significantly only in the PPI-1011 50 mg/kg treatment. The MPTP lesion led to elevated HVA/DA ratios (Fig 1G) that remained elevated in mice that also received the PPI-1011 treatments. The 3-MT/DA (Fig 1F) and DOPAC/DA (Fig 1E) ratios remained unaffected by the MPTP lesion and the PPI-1011 treatments except for 3-MT/DA that was elevated in the MPTP + PPI-1011 50 mg/kg group (Fig 1F).

**Fig 2:**
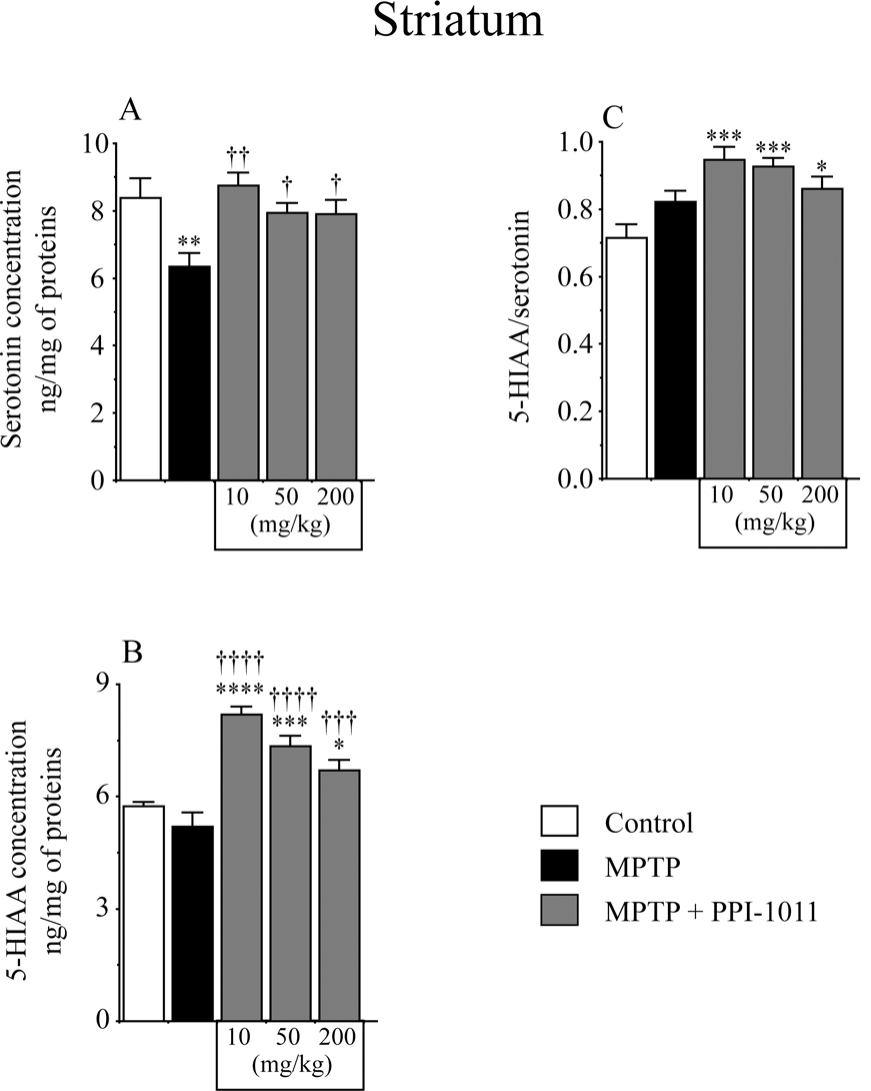
PPI-1011 pre-treatment neuroprotection of striatal serotonin and its metabolite. Effect of MPTP and PPI-1011 treatment on striatal A) serotonin (*F*_4,46_ = 4.04, *p* = 0.007) contents and its metabolite B) 5-hydroxyindoleacetic acid (5-HIAA) (*F*_4,46_ = 19.83, *p* < 0.0001), as well as C) 5-HIAA/serotonin (*F*_4,46_ = 7.04, *p* = 0.0002) ratio. Values shown are the means (ng/mg of proteins) ± S.E.M. of 9–12 mice per group. * p < 0.05, ** p < 0.01, *** p < 0.001 and **** p < 0.0001 vs control; † p < 0.05, †† p < 0.01 ††† p < 0.001 and †††† p < 0.0001 vs MPTP.

Pre-treatment with PPI-1011, at all doses tested, prevented the small MPTP-induced loss of striatal serotonin contents (Fig 2A). MPTP alone did not affect striatal 5-HIAA contents (Fig 2B) nor the 5-HIAA/serotonin ratios (Fig 2C), however MPTP + PPI-1011 treatments at all doses significantly increased 5-HIAA levels and the 5-HIAA/serotonin ratios (Fig 2B and 2C).

**Fig 3:**
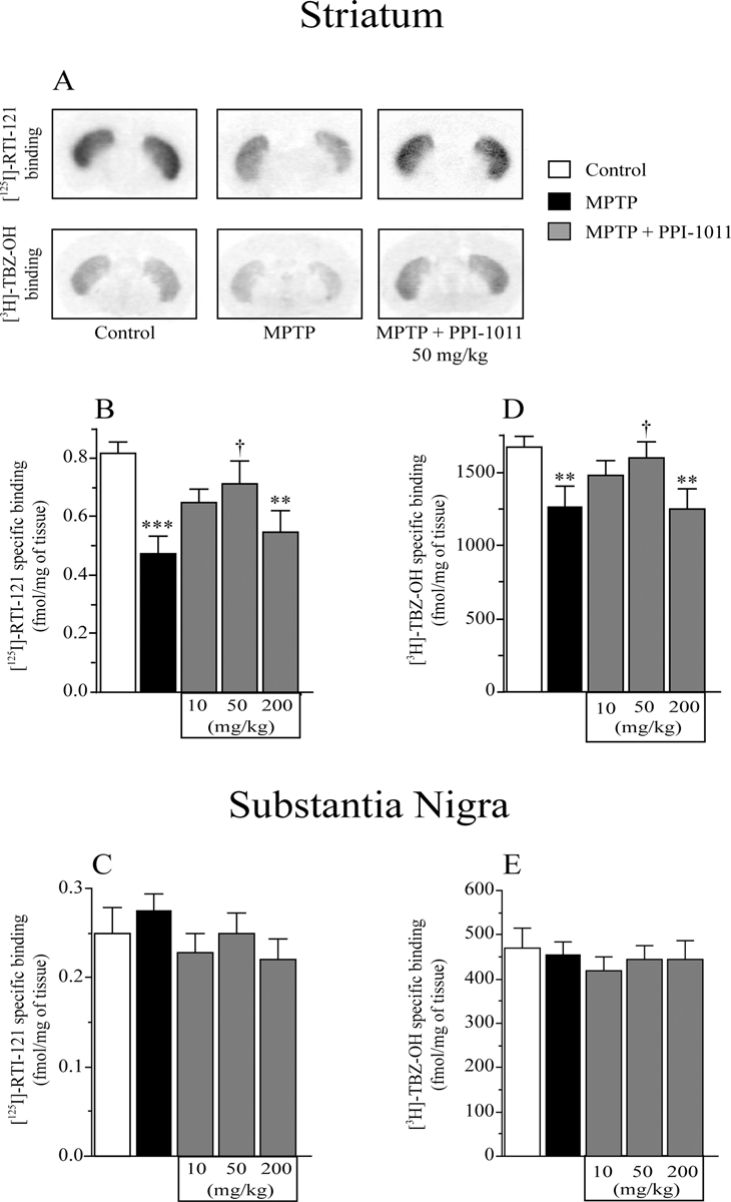
PPI-1011 pre-treatment neuroprotection of nigrostriatal dopamine transporters. Examples A) and effect of MPTP and PPI-1011 treatment in mice on DAT ([^125^I]-RTI) specific binding in B) striatum (*F*_4,44_ = 5.51, *p* = 0.001) and C) substantia nigra (*F*_4,44_ = 1.15, *p* = 0.35) as well as VMAT2 ([^3^H]-TBZ-OH) specific binding in D) striatum (*F*_4,44_ = 4.05, *p* = 0.007) and E) substantia nigra (*F*_4,44_ = 0.24, *p* = 0.91). Values shown are the means (fmol/mg of tissue) ± S.E.M. of 9–12 mice per group. ** p < 0.01 and *** p < 0.001 vs control; † p < 0.05 vs MPTP.

Autoradiography analysis of the binding capacity of DAT and VMAT2 was performed in striatum and substantia nigra samples from the pre-treatment experiment (Fig 3A). MPTP led to significant decreases of DAT ([^125^I]-RTI-121) (Fig 3B) and VMAT2 ([^3^H]-TBZ-OH) (Fig 3D) specific binding in the striatum. PPI-1011 pre-treatment prevented DAT and VMAT2 specific binding loss caused by the MPTP lesion. Treatment with PPI-1011 at 10 mg/kg was partially protective, while 50 mg/kg maintained control binding levels. Further elevation to 200 mg/kg PPI-1011 lead to a loss of protection, with binding efficiencies similar to those seen in the MPTP alone treated animals. Neither MPTP nor PPI-1011 treatments altered DAT or VMAT2 specific binding in the substantia nigra (Fig 3C and 3E).

**Fig 4:**
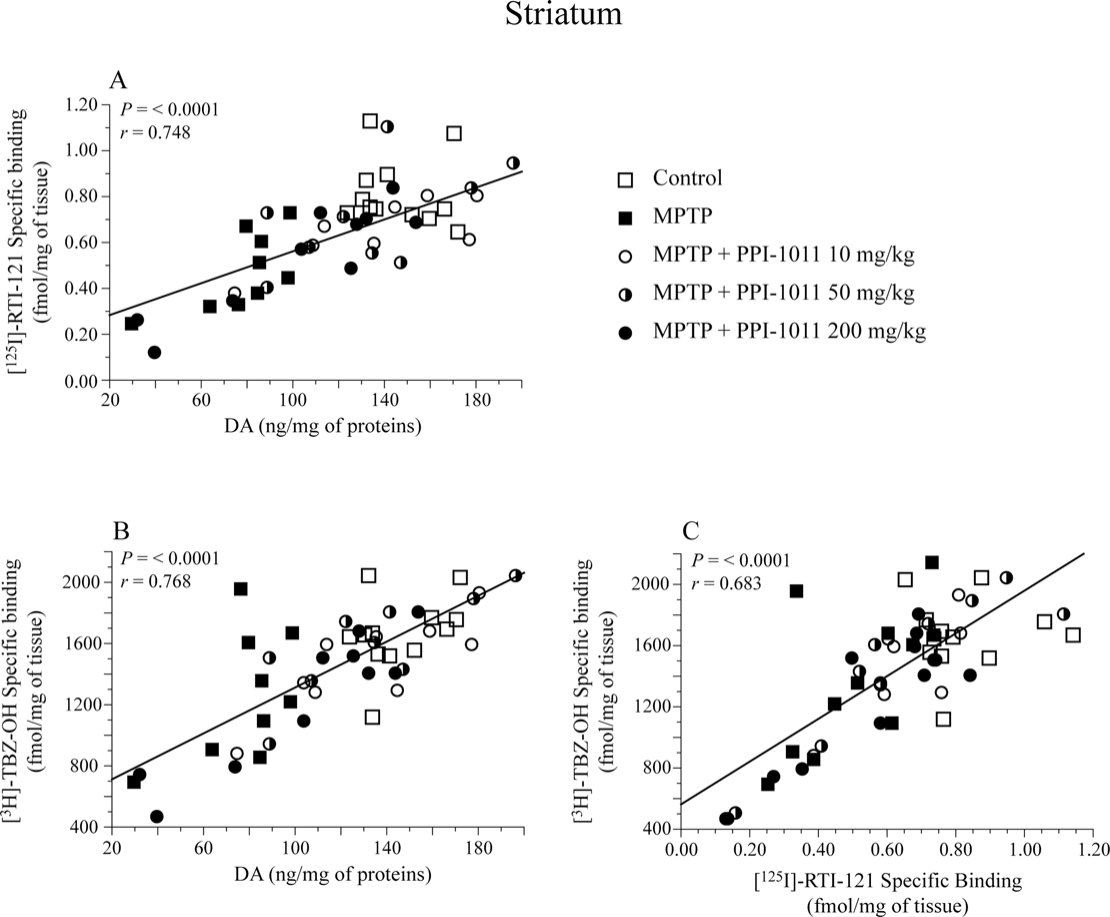
Correlation of striatal dopamine markers in PPI-1011 pre-treatment neuroprotection. Correlations between dopamine (DA) contents and A) DAT and B) VMAT2 specific binding, as well as C) between DAT and VMAT2 specific binding in mice treated with MPTP and PPI-1011.

Significant positive correlations were observed between striatal DA content and both DAT and VMAT2 specific binding in the PPI-1011 pre-treatment experiment (Fig 4A and 4B). Moreover, a significant positive correlation was also observed between DAT and VMAT2 specific binding in the striatum (Fig 4C).

**Fig 5:**
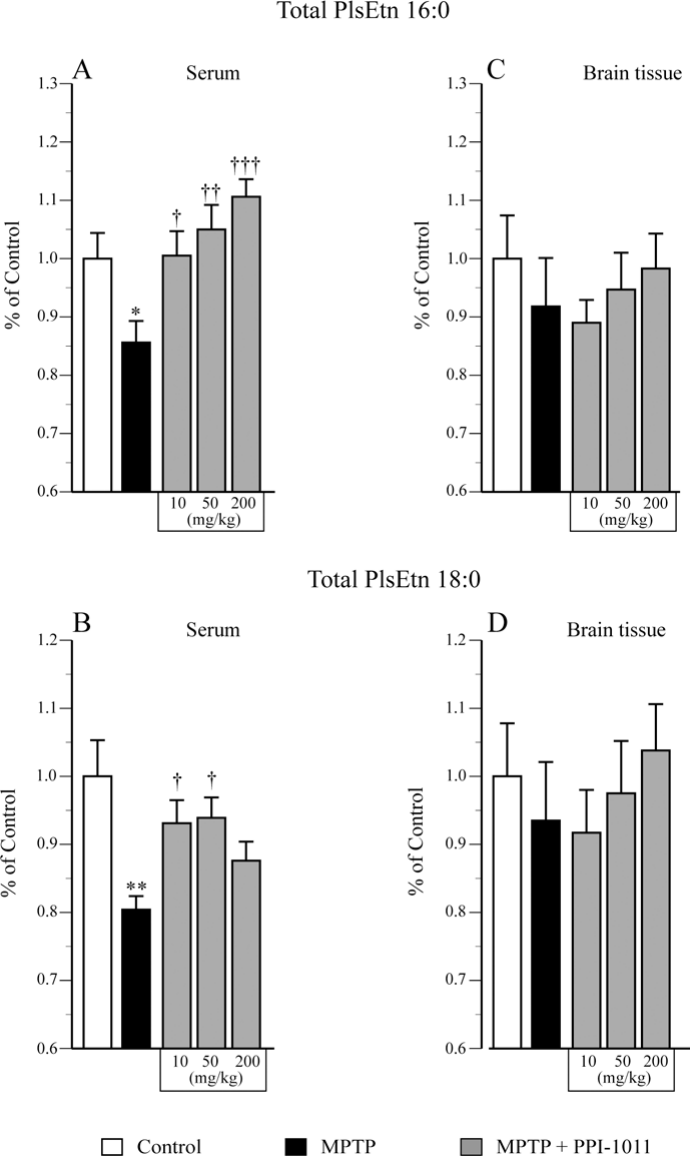
PPI-1011 pre-treatment neuroprotection of serum and brain plasmalogen levels. Effect of MPTP and PPI-1011 treatment in mice on total serum levels of A) 16:0 plasmalogens (*F*_4,46_ = 5.29, *p* = 0.001) and B) 18:0 plasmalogens (*F*_4,46_ = 4.15, *p* = 0.006) as well as in brain tissue of C) 16:0 plasmalogens (*F*_4,53_ = 0.78, *p* = 0.78) and D) 18:0 plasmalogens (*F*_4,53_ = 0.40, *p* = 0.89). Values shown are the means (relative to controls) ± S.E.M. of 9–12 mice per group. * p < 0.05 and ** p < 0.01 vs control; † p < 0.05, †† p < 0.01and ††† p < 0.001 vs MPTP.

PPI-1011 was designed to be converted into PlsEtn 16:0/22:6 following addition of the ethanolamine head group and formation of the vinyl ether bond *in vivo*. While the target was PlsEtn 16:0/22:6, rearrangement into a variety of PlsEtn species is known to occur [9]. The majority of PlsEtn species contains 16:0 or 18:0 as the sn-1 constituent, with a variety of fatty acids located at sn-2. In addition to our analysis of individual PlsEtn species, we analyzed the 16:0 and 18:0 PlsEtn pools to account for changes in total PlsEtn levels. MPTP treatment decreased serum concentrations of 16:0 and 18:0 PlsEtn (Fig 5A and 5B). Total serum 16:0 PlsEtn concentrations dose-dependently increased with PPI-1011 treatment compared to MPTP treated mice, to levels at or above control. In contrast, 18:0 PlsEtns levels were elevated only with PPI-1011 10 and 50 mg/kg treatments (Fig 5B). A similar pattern for both 16:0 and 18:0 PlsEtn was observed in brain tissue (including the cerebellum) of these mice (Fig 5C and 5D). Levels were reduced following MPTP lesion while PPI-1011 treatment prevented this decline; however, these changes did not reach statistical significance (Fig 5C and 5D).

**Fig 6:**
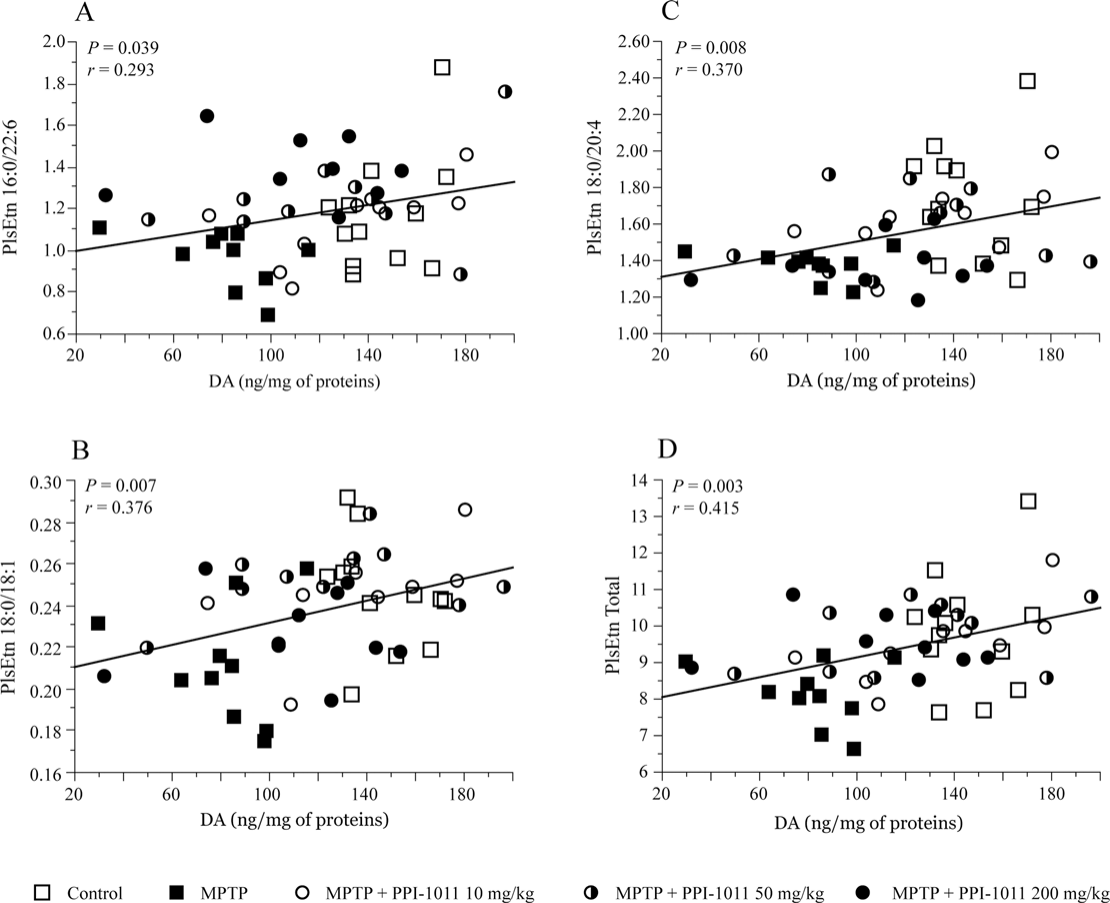
Correlation of striatal dopamine concentrations and plasmalogen levels in PPI-1011 pre-treatment neuroprotection. Correlations between the levels of serum plasmalogens A) 16:0/22:6, B) 18:0/18:1 and C) 18:0/20:4 and striatal dopamine (DA) concentrations as well as D) correlation between total serum plasmalogen levels and DA content.

Positive correlations between striatal DA contents and serum levels of the target PlsEtn (16:0/22:6) as well as with PlsEtn 18:0/18:1 and PlsEtn 18:0/20:4 were observed (Fig 6A, 6B and 6C). We assessed the correlation of PlsEtn 18:0/18:1 and PlsEtn 18:0/20:4 with DA in addition to the target PlsEtn 16:0/22:6 because in addition to DHA, oleic acid (18:1) and arachidonic acid (20:4) containing PlsEtn are the most abundant in the brain. The total sum of serum PlsEtns measured also correlated with striatal DA contents (Fig 6D).

**Fig 7:**
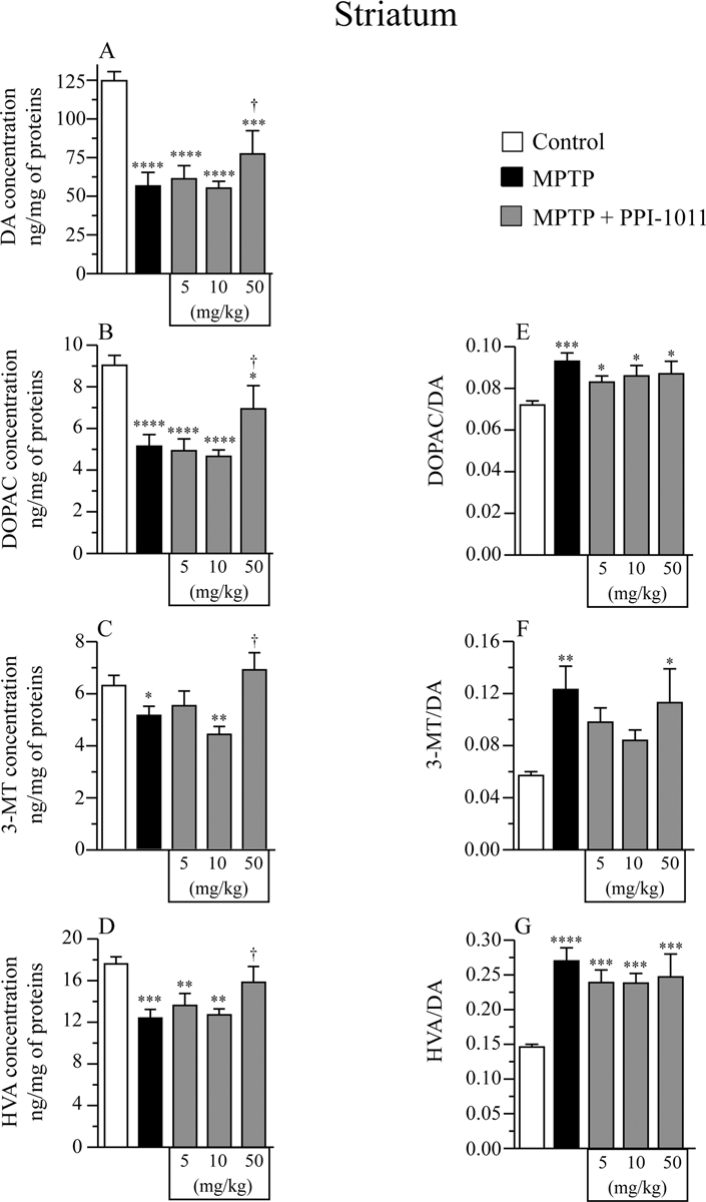
PPI-1011 post-treatment neuroprotection of striatal dopamine and its metabolites. Effect of MPTP and PPI-1011 treatment on striatal A) DA contents (*F*_4,49_ = 15.76, *p* < 0.0001) and its metabolites B) DOPAC (*F*_4,49_ = 10.53, *p* < 0.0001), C) 3-MT (*F*_4,49_ = 5.59, *p* = 0.0009) and D) HVA (*F*_4,49_ = 6.20, *p* = 0.0004), as well as E) DOPAC/DA (*F*_4,49_ = 4.87, *p* = 0.002), F) 3-MT/DA (*F*_4,49_ = 3.92, *p* = 0.008) and G) HVA/DA (*F*_4,49_ = 8.60, *p* < 0.0001) ratios. Values shown are the means (ng/mg of proteins) ± S.E.M. of 9–14 mice per group. * p < 0.05, ** p < 0.01, *** p < 0.001 and **** p < 0.0001 vs control; † p < 0.05 vs MPTP.

In the post-treatment experiment, MPTP induced a decrease of striatal DA contents that was prevented by PPI-1011 treatment at 50 mg/kg, while the lower doses of 5 and 10 mg/kg were not protective (Fig 7A). Moreover, PPI-1011 treatment at 50 mg/kg prevented the MPTP-induced decrease of striatal metabolites DOPAC, 3-MT and HVA (Fig 7B, 7C and 7D) contents while no effect was observed using the lower doses. The MPTP lesion and PPI-1011 treatments similarly increased the striatal DOPAC/DA, 3-MT/DA and HVA/DA ratios (Fig 7E, 7F and 7G), except for the 3-MT/DA ratio that was not increased in MPTP mice treated with 5 and 10 mg/kg PPI-1011 (Fig 7F).

**Fig 8:**
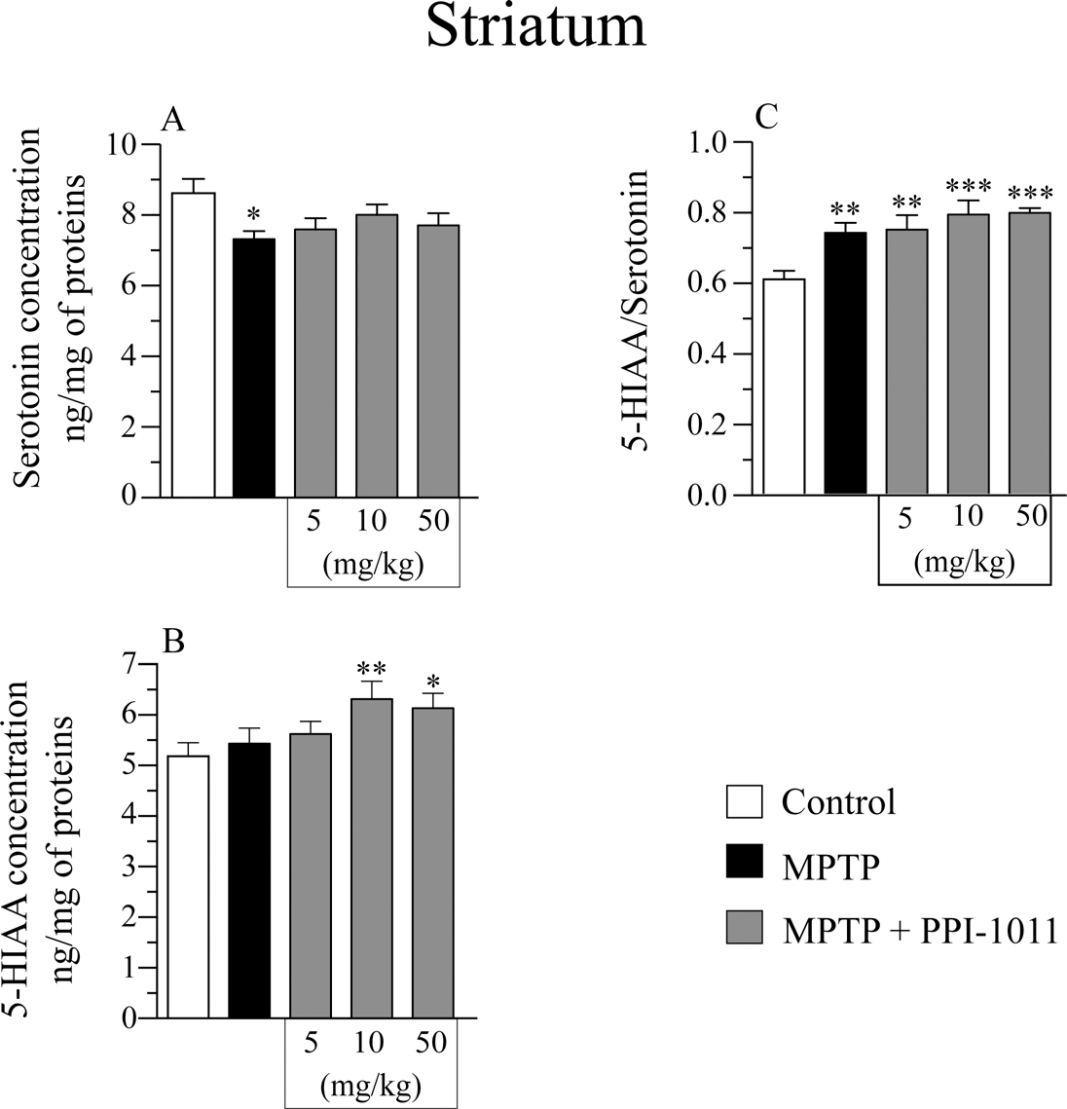
PPI-1011 post-treatment neuroprotection of striatal serotonin and its metabolite. Effect of MPTP and PPI-1011 treatment on striatal A) serotonin (*F*_4,49_ = 2.66, *p* = 0.04) contents and its metabolite B) 5-hydroxyindoleacetic acid (5-HIAA) (*F*_4,49_ = 3.52, *p* = 0.01), as well as C) 5-HIAA/serotonin (*F*_4,49_ = 7.05, *p* = 0.0001) ratio. Values shown are the means (ng/mg of proteins) ± S.E.M. of 9–14 mice per group. * p < 0.05, ** p < 0.01 and *** p < 0.001 vs control.

Striatal serotonin (Fig 8A) contents decreased with the MPTP lesion while 5-HIAA contents (Fig 8B) remained unchanged in the post-treatment experiment. PPI-1011 at all doses tested left unchanged striatal serotonin contents while 5-HIAA levels increased with PPI-1011 10 and 50 mg/kg. (Fig 8B). All MPTP lesioned mice showed an increase in 5-HIAA/serotonin ratios and this augmentation was similar in PPI-1011 treated mice (Fig 8C).

**Fig 9:**
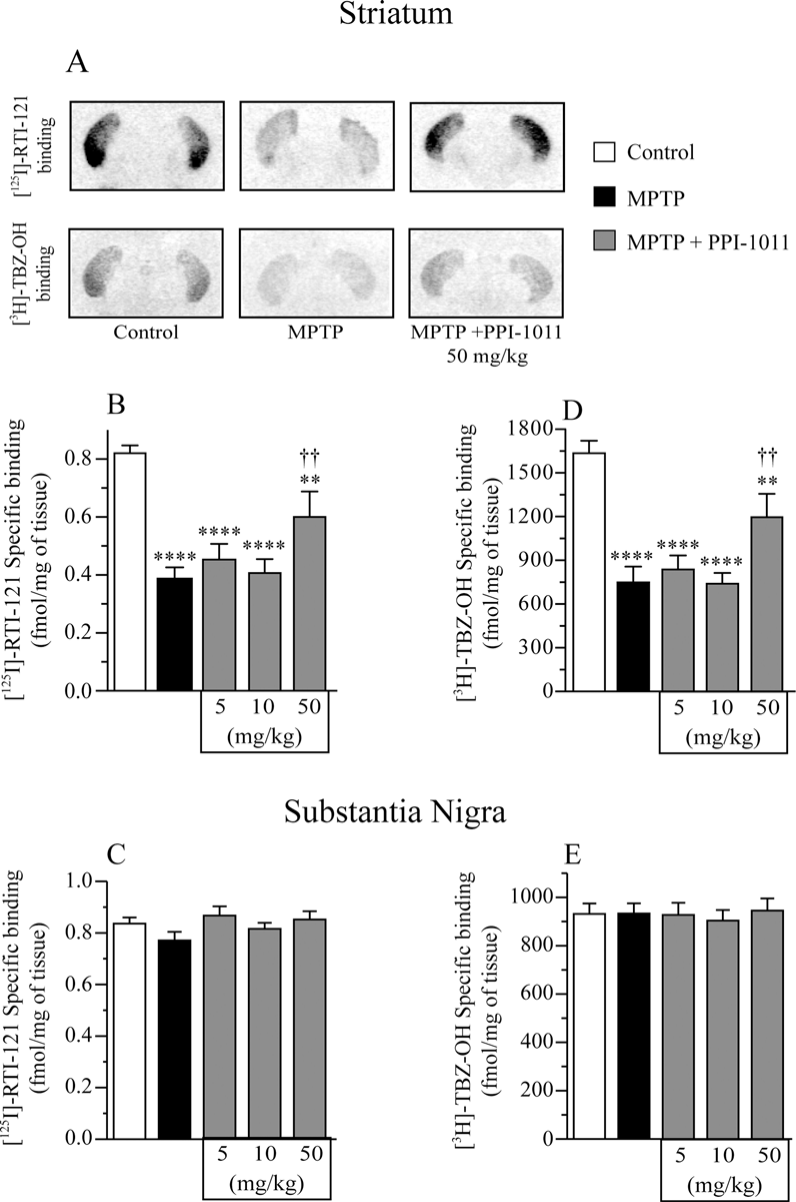
PPI-1011 post-treatment neuroprotection of nigrostriatal dopamine transporters. Examples A) and effect of MPTP and PPI-1011 treatment in mice on DAT ([^125^I]-RTI) specific binding in B) striatum (*F*_4,46_ = 15.17, *p* <0.0001) and C) substantia nigra (*F*_4,49_ = 1.55, *p* = 0.20) as well as VMAT2 ([^3^H]-TBZ-OH) specific binding in D) striatum (*F*_4,48_ = 16.44, *p* < 0.0001) and E) substantia nigra (*F*_4,47_ = 0.27, *p* = 0.90). Values shown are the means (fmol/mg of tissue) ± S.E.M. of 8–14 mice per group. ** p < 0.01, *** p < 0.001 and **** p < 0.0001 vs control; †† p < 0.01 vs MPTP.

Autoradiography from the post-treatment experiment in striatum and substantia nigra (Fig 9A) revealed that MPTP led to a decrease of DAT and VMAT2 specific binding in the striatum (Fig 9B and 9D), while no changes were seen in the substantia nigra (Fig 9C and 9E). PPI-1011 treatment at 50 mg/kg rescued the DAT and VMAT2 specific binding decreases observed in MPTP mice while lower doses were ineffective.

**Fig 10:**
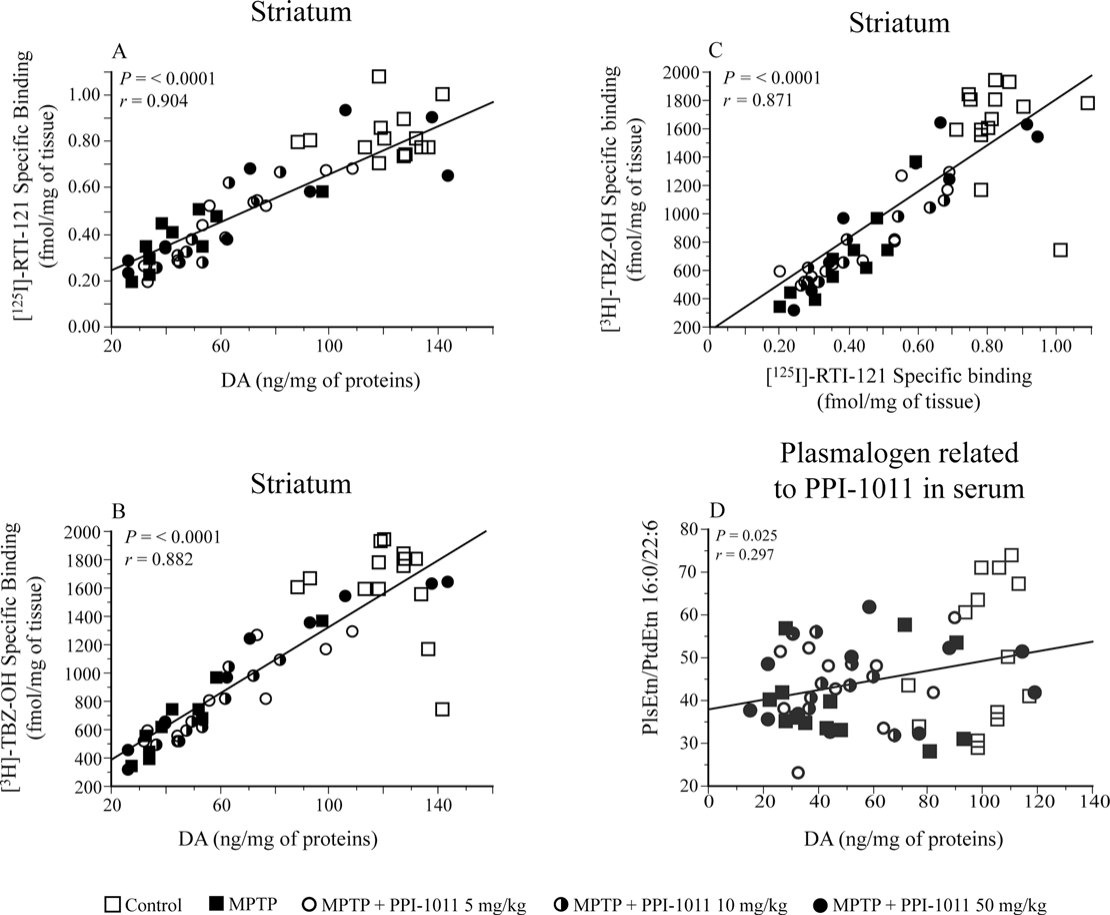
Correlation of striatal dopamine markers and plasmalogen in PPI-1011 post-treatment neuroprotection. Correlations between dopamine (DA) contents and A) DAT and B) VMAT2 specific binding, C) between DAT and VMAT2 specific binding in mice treated with MPTP and PPI-1011 as well as correlation D) between the serum level of plasmalogen 16:0/22:6 in serum and striatal DA contents.

Significant positive correlations between striatal DA contents and both DAT and VMAT2 specific binding were observed in the post-treatment experiment (Fig 10A and 10B). Moreover, a significant positive correlation was also observed between striatal DAT and VMAT2 specific binding in this experiment (Fig 10C).

As seen in the pre-treatment experiment there was a positive correlation between the serum levels of the target PlsEtn (16:0/22:6) and DA contents in the post-treatment experiment (Fig 10D) whereas the correlation between the 18:0 PlsEtn and DA content was not significant (R = 0.21, p = 0.11).

The doses of PPI-1011 investigated against MPTP toxicity that is at 5, 10 and 50 mg/kg were tested in two separate experiments in intact male mice administered once daily for 10 days. That is the same administration protocol but in unlesioned (absence of MPTP) mice. The highest dose of PPI-1011 of 200 mg/kg was not tested in intact mice since it did not show neuroprotection of dopamine markers. PPI-1011 did not change striatal biogenic amines levels in intact mice (Table 1); considering this lack of effect, other DA markers or PlsEtns levels were not measured in these mice.

**Table 1:** Effect of PPI-1011 treatment for 10 days of intact mice on striatal biogenic amine contents.

Biogenic amines measured	Control	PPI-1011		
		5 mg/kg	10 mg/kg	50 mg/kg
	(% of control values)			
DA	100.0 ± 3.1	105.4 ± 3.9	103.6 ± 3.7	99.2 ± 6.1
DOPAC	100.0 ± 3.5	107.7 ± 6.8	93.9 ± 3.6	98.6 ± 6.9
3-MT	100.0 ± 8.4	135.2 ± 10.2	118.3 ± 15.1	79.4 ± 10.2
HVA	100.0 ± 5.4	94.6 ± 3.0	101.3 ± 3.2	103.9 ± 8.1
serotonin	100.0 ± 3.3	117.3 ± 6.6	118.5 ± 3.1	104.8 ± 6.1
5HIAA	100.0 ± 5.0	80.8 ± 3.4	102.3 ± 5.7	109.7 ± 10.1

100% control values from two separate experiments were for DA: 140.0; DOPAC: 9.6; 3-MT: 5.6; HVA: 15.2; serotonin: 3.9 and 5-HIAA: 4.5 ng/mg proteins. No statistical differences between these experimental groups were observed.

## Discussion

Brain contains the highest amounts of tissue PlsEtns and their reduction can be demonstrated in various neurodegenerative diseases including Alzheimer’s disease and PD (reviewed in [2]). It is unclear whether PlsEtn loss is a contributing cause or downstream effect of pathology. PPI-1011 an orally bioavailable PlsEtn precursor analog, was prepared [27] to test whether PlsEtn replacement therapy was beneficial in neurodegenerative disorders.

Although PlsEtn are abundant in humans, their physiological roles have been challenging to identify likely due to the specificity of these roles in different tissues, metabolic processes and development stages [2]. Considerable refinements have been made in the methodology employed to assay tissue PlsEtn over the past few decades [2], allowing more robust analysis. While an earlier study found no change of membrane PlsEtn in the substantia nigra of five PD patients compared to controls [28], more recent studies have reported decreased serum concentrations of PlsEtn in patients with PD which was suggested to be a marker of systemic oxidative stress [6]. An extensive decrease of PlsEtn concentrations was also reported in the frontal cortex lipid rafts of PD patients [7].

The present studies are the first to report decreased serum PlsEtn in MPTP treated mice, supporting the relevance of this PD model to investigate PlsEtn neuroprotection. We observed a decrease in serum PlsEtn concentrations in the MPTP lesioned mice that were prevented with PlsEtn precursor treatment. These results allowed us to conclude that the endogenous synthesis capacity of essential membrane PlsEtn was insufficient to meet the increased demand required to maintain membrane integrity in this neurotoxic model of PD and that this insufficiency contributes to the observed neurodegeneration. A similar pattern of changes in PlsEtn concentrations was observed in the brain and in the serum but the former did not reach statistical significance. PlsEtn effects localized to the lesion may have been diluted by the surrounding normal brain tissue affecting the assay.

Supporting the plasmalogen augmentation with PPI-1011, we previously showed that PPI-1011 raises PlsEtn levels in cell culture, rabbit and mouse plasma and tissues including the brain. Dose-dependent augmentation of DHA-PlsEtn levels, in wild type CHO cells were observed at 1–20 μM of PPI-1011 as well as restoration of PlsEtn levels in PlsEtn-deficient cell lines (NRel-4) [27]. Incorporation of labelled compound into circulating PlsEtn 16:0/22:6 was observed with a stable isotope analogue of PPI-1011 (100 mg/kg) in rabbits; and incorporation of the labelled components in to PlsEtn 16:0/22:6 were also seen in the brain [9]. Labelling of PlsEtn 16:0/22:6 pool in adrenal, kidney, liver, neocortex and eyes by three-day treatment of stable isotope analogue of PPI-1011 (100 mg/kg) was observed in PEX7 murine model of rhizomelic chrondrodysplasia punctate-1 [8]. These experiments show the proof of concept that PPI-1011 can augment circulating levels and gets incorporated into the brain as well. Demonstration of the localized elevation of PlsEtn in the mouse striatum and substantia nigra would have been very convincing but with existing technology, it is difficult to extract and quantify PlsEtn 16:0/22:6 in micro-quantities of brain tissue.

Our results showed that PPI-1011 protected striatal DA against MPTP toxicity in mice. The neuroprotective effect of the PlsEtn precursor appeared to have a bell-curve dose-dependency in that the effect was reduced at the highest dose tested. Hence, our pre-MPTP PPI-1011 administrations showed that PPI-1011 treatment protected against MPTP toxicity at 10 and 50 mg/kg and the post-MPTP PPI-1011 administrations protected against MPTP toxicity at 50 mg/kg. This suggests that in PD patients a PPI-1011 treatment could be beneficial even at lower doses.

DHA has previously been reported to protect DA neurons against MPTP toxicity in mice [3]. Hence, the neuroprotection activity of the DHA containing PlsEtn precursor (PPI-1011) observed here could result from augmentation of either the PlsEtn backbone or DHA. However, considering the positive correlation of blood PlsEtn and striatal DA levels, studies utilizing other phospholipid containing PlsEtn precursors will help to clarify the structure activity relationship of PlsEtn neuroprotection.

An extensive loss of striatal DA is well documented in PD but other neurotransmitters such as serotonin are also decreased to a lesser extent [29–31]. The present experiments using MPTP doses to obtain a moderate loss of striatal DA contents modeling early stages of PD as expected led to a small decrease of striatal serotonin content. Interestingly, all doses of PPI-1011 administered for 10 days prevented the MPTP-induced loss of serotonin whereas this was not observed in the post-treatment experiment that was of a shorter duration of 5 days. Moreover, increase levels of the serotonin metabolite 5-HIAA and turnover as estimated with the 5-HIAA/serotonin ratio was observed. This suggests serotonin neuroprotection and increased activity in the striatum. This effect of PPI-1011 may bring an added benefit in PD neuroprotection considering that depression is a common and important non-motor aspect of PD and that selective serotonin uptake inhibitor significantly improve depression in PD patients (systematic review and meta-analysis: [32]).

PlsEtn are considered sensitive markers of oxidative stress [33] and liver is the source of circulating PlsEtn [9]. Therefore, MPTP-induced reductions of 16:0 and 18:0 PlsEtn pool, could possibly be due to the oxidative stress induced by MPTP or can also be due to the possible hepatotoxic effects of MPTP [34]. Treatment with PPI-1011 had the anticipated effect on the 16:0 PlsEtn pool, with levels increasing in a dose-dependent manner. The same trend was not observed in the 18:0 pool, with elevated levels only in the two lower dose groups. Although PPI-1011 was a precursor to PlsEtn 16:0/22:6, an array of plasmalogen species gets elevated as a result of enzymatic remodelling. This results in an increase in both 16:0 and 18:0 (sn-1) plasmalogen pool, as we have seen in this study. This is important in the context of membrane composition where 16:0 and 18:0 pools are present approximately at equal abundance [35]. This is also true for the brain as well [25]. Therefore, maintenance of this equilibrium is probably important for optimal biological function, particularly the membrane function. The 200 mg/kg group however had lower 18:0 PlsEtn levels, presumably due to an equilibrium shift, which might have led to the less than optimal biological activity at this dose. The goal of PPI-1011 is to support peroxisomal function and maintain endogenous PlsEtn levels, in spite of a neurotoxic insult. The 200 mg/kg dose may be excessive, shifting the equilibrium of PlsEtn species, resulting in changes that hamper its neuroprotective abilities in response to MPTP treatment.

DA metabolism has been shown to involve a functional and physical protein complex of VMAT2, tyrosine hydroxylase (TH) and aromatic amino acid decarboxylase (AADC) located at the synaptic vesicle membrane [36]. TH converts tyrosine to L-DOPA that is further converted to DA by AADC. Additionally, AADC also converts 5-hydroxy-L-tryptophan into serotonin. The newly synthesized monoamines, DA and serotonin, are then loaded into synaptic vesicles by VMAT2, ensuring that cytosolic monoamine levels remain low. MPTP-induced reductions in DA (and its metabolites) as well as serotonin levels and VMAT2 binding suggest that MPTP alters the function of the components of this complex. DAT functions to transport DA from outside the synapse back into the cytosol, which is then re-packaged into vesicles by VMAT2. Alterations in the PlsEtn membrane composition have been shown to dramatically alter the function of membrane proteins [37, 38]. Decreases in membrane PlsEtn levels also lead to altered cholesterol transport [27, 39–41] that can have drastic effects on membrane-associated functions. Indeed, depletion of total membrane cholesterol with methyl-ß-cyclodextrin (mßCD) was reported to reduce both DA uptake and efflux rate, showing the importance of cholesterol-DAT interactions in regulating DAT function [42]. Therefore, the restoration of membrane equilibrium by PPI-1011 can be speculated to restore imbalances in DA recycling.

Reductions in PlsEtn levels in response to neurotoxic insults, such as MPTP, could alter the dynamics of the TH, AADC, VMAT2 complex leading to reduction in DA synthesis and transport of DA and serotonin into vesicles. These reductions could also affect the function of DAT. Since critical steps in plasmalogen biosynthesis need peroxisomes, which can become dysfunctional due to age and other factors, exogenous supply of plasmalogen precursors that bypass peroxisomal steps in fact support peroxisomal function, and help maintain PlsEtn levels in blood and tissues [8, 9] and assist in retaining optimal membrane function [27]. All of these could contribute to the prevention or restoration of MPTP-induced neuronal dysfunction observed in the current study. However, another possibility is that PPI-1011 prevents MPTP induced toxicity by a direct interaction. A limitation of this study was that we did not test the effect of PPI-1011 on the dynamics of TH, AADC and VMAT2 complex. However, it is doubtful whether PPI-1011 will affect these functions in the intact animal since the membrane equilibrium, which is the putative mechanism of action of PPI-1011, will be intact in these animals. Indeed, we observed no effect of PPI-1011 treatment on striatal biogenic amines of intact mice.

Hence, PlsEtn might be altering the function of membrane proteins such as DAT/VMAT2 and neurotransmitter levels in order to afford neuroprotection. But these transporters and neurotransmitters are affected by MPTP itself, so the preservation of these markers might also reflect the protection against MPTP’s actions, rather than highlighting the mechanism responsible for the protection itself. Both functional and neuroprotective activities of PlsEtn may be occurring since in PPI-1011 MPTP treated mice increases are observed compared to MPTP alone and in some case, for metabolites, compared to controls.

The neuroprotection of PPI-1011 on striatal DA contents in response to MPTP toxicity could also involve anti-apoptotic mechanisms. A recent study reported that PlsEtn rescued neuronal cell death caused by serum deprivation through activation of Akt and ERK survival signalling [43] and we [44] as well as several investigators (reviewed in [20]) have previously shown neuroprotection of DA neurons against MPTP toxicity by hormones known to exert effects on these anti-apoptotic proteins.

Numerous compounds, of different chemical classes, have shown neuroprotective activity in various animal models of PD but fewer compounds have been reported to rescue nigrostriatal DA against injuries or toxicities. Mono-target drugs may not possess disease-modifying activity in progressive neurodegenerative disease such as PD that has complex pathology and molecular cascade of events. Thus multi–target drugs may better restore and rescue neurons in PD [45, 46]. PPI-1011, as a precursor of plasmalogens with various activities, could be considered as a multi-target drug.

## Conclusion

As observed in PD [6], MPTP treated mice showed reductions in circulating PlsEtn levels, highlighting its usefulness as a model to study the effect of PlsEtn replenishment on brain DA neurotransmission. The present results are the first to show neuroprotection activity of an oral plasmalogen replenishment therapy on striatal DA markers in an animal model of PD. PPI-1011 is designed such that it is metabolized into naturally occurring entities prior to entering circulation. The endogenous nature of the metabolites of PPI-1011 should result in a favourable safety profile, allowing for ease of translation into humans with PD; the goal of treatment being preventing, stopping or slowing disease progression.

## Supporting Information

S1 File Table A. List of analytes measured in serum and Table B. List of analytes measured in brain tissue.
(DOCX)

## Abbreviations

AADC: aromatic amino acid decarboxylase
BBB: blood-brain barrier
DA: dopamine
DAT: dopamine transporter
DHA: docosahexaenoic acid
DOPAC: 3,4-dihydroxyphenylacetic
HPLC: high-performance liquid chromatography
HVA: homovanillic acid
MPTP: 1-methyl-4-phenyl-1,2,3,6-tetrahydropyridine
MS: mass spectrometry
PD: Parkinson’s disease
PlsEtn: plasmalogen ethanolamine
PtdEtn: Phosphatidylethanolamine
TH: tyrosine hydroxylase
VMAT2: vesicular monoamine transporter 2
