# Supplementary material for: Plasmalogen Augmentation Reverses Striatal Dopamine Loss in MPTP Mice
Source: PLoS One. 2016 Mar 9;11(3):e0151020. doi: 10.1371/journal.pone.0151020 (PMC4784967; doi:10.1371/journal.pone.0151020)
Supplement: S1 File — Table A. List of analytes measured in serum and Table B. List of analytes measured in brain tissue. (DOCX) [file pone.0151020.s001.docx]

**Dopaminergic protection by plasmalogen augmentation in parkinsonian mice**

*Edith-Miville Godbout, Mélanie Bourque, Marc Morissette, Sara Al-Sweidi, Tara Smith, Asuka Mochizuki, Vijitha Senanayake, Dushmanthi Jayasinghe, Li Wang,*

*Dayan Goodenowe and Thérèse Di Paolo*

Table A. List of analytes measured in serum.

| Analyte | Molecular Formula | MRM^a^ transition | Analyte | Molecular Formula | MRM^a^ transition |
| --- | --- | --- | --- | --- | --- |
| ^13^C-PtdEtn 16:0/22:6 | C_24_^13^C_19_H_74_NO_8_P | 781.5/327.2 | ^13^C-PlsEtn 16:0/22:6 | C_37_^13^C_6_H_74_NO_7_P | 752.5/327.2 |
| PtdEtn 16:0/18:0 | C_39_H_78_NO_8_P | 718.5/255.2 | PlsEtn 16:0/18:0 | C_39_H_78_NO_7_P | 702.5/283.2 |
| PtdEtn 16:0/18:1 | C_39_H_76_NO_8_P | 716.5/255.2 | PlsEtn 16:0/18:1 | C_39_H_76_NO_7_P | 700.5/281.2 |
| PtdEtn 16:0/18:2 | C_39_H_74_NO_8_P | 714.5/255.2 | PlsEtn 16:0/18:2 | C_39_H_74_NO_7_P | 698.5/279.2 |
| PtdEtn 16:0/18:3 | C_39_H_72_NO_8_P | 712.5/255.2 | PlsEtn 16:0/18:3 | C_39_H_72_NO_7_P | 696.5/277.2 |
| PtdEtn 16:0/20:4 | C_41_H_74_NO_8_P | 738.5/255.2 | PlsEtn 16:0/20:4 | C_41_H_74_NO_7_P | 722.5/303.2 |
| PtdEtn 16:0/22:4 | C_43_H_78_NO_8_P | 766.5/255.2 | PlsEtn 16:0/22:4 | C_43_H_78_NO_7_P | 750.5/331.2 |
| PtdEtn 16:0/22:6 | C_43_H_74_NO_8_P | 762.5/255.2 | PlsEtn 16:0/22:6 | C_43_H_74_NO_7_P | 746.5/327.2 |
| PtdEtn 16:0/24:6 | C_45_H_78_NO_8_P | 790.5/255.2 | PlsEtn 18:0/18:0 | C_41_H_82_NO_7_P | 730.5/283.2 |
| PtdEtn 16:0/28:0 | C_49_H_98_NO_8_P | 858.7/255.2 | PlsEtn 18:0/18:1 | C_41_H_80_NO_7_P | 728.5/281.2 |
| PtdEtn 18:0/18:0 | C_41_H_82_NO_8_P | 746.5/283.2 | PlsEtn 18:0/18:2 | C_41_H_78_NO_7_P | 726.5/279.2 |
| PtdEtn 18:0/18:1 | C_41_H_80_NO_8_P | 744.5/283.2 | PlsEtn 18:0/18:3 | C_41_H_76_NO_7_P | 724.5/277.2 |
| PtdEtn 18:0/18:2 | C_41_H_78_NO_8_P | 742.5/283.2 | PlsEtn 18:0/20:4 | C_43_H_78_NO_7_P | 750.6/303.2 |
| PtdEtn 18:0/18:3 | C_41_H_76_NO_8_P | 740.5/283.2 | PlsEtn 18:0/22:4 | C_45_H_82_NO_7_P | 778.5/331.2 |
| PtdEtn 18:0/20:4 | C_43_H_78_NO_8_P | 766.5/283.2 | PlsEtn 18:0/22:6 | C_45_H_78_NO_7_P | 774.5/327.2 |
| PtdEtn 18:0/22:4 | C_45_H_82_NO_8_P | 794.5/283.2 |  |  |  |
| PtdEtn 18:0/22:6 | C_45_H_78_NO_8_P | 790.5/283.2 |  |  |  |
| PtdEtn 18:0/24:6 | C_47_H_82_NO_8_P | 818.5/283.2 |  |  |  |
| PtdEtn 18:0/28:0 | C_51_H_102_NO_8_P | 886.7/283.2 |  |  |  |

a) MRM: multiple reaction monitoring

Table B. List of analytes measured in brain tissue.

| Analyte | Molecular Formula | MRM^a^ transition | Analyte | Molecular Formula | MRM^a^ transition |
| --- | --- | --- | --- | --- | --- |
| ^13^C-PtdEtn 16:0/22:6 | C_24_^13^C_19_H_74_NO_8_P | 781.5/327.2 | ^13^C-PlsEtn 16:0/22:6 | C_37_^13^C_6_H_74_NO_7_P | 752.5/327.2 |
| PtdEtn 16:0/18:0 | C_39_H_78_NO_8_P | 718.5/255.2 | PlsEtn 16:0/18:0 | C_39_H_78_NO_7_P | 702.5/283.2 |
| PtdEtn 16:0/18:1 | C_39_H_76_NO_8_P | 716.5/255.2 | PlsEtn 16:0/18:1 | C_39_H_76_NO_7_P | 700.5/281.2 |
| PtdEtn 16:0/18:2 | C_39_H_74_NO_8_P | 714.5/255.2 | PlsEtn 16:0/18:2 | C_39_H_74_NO_7_P | 698.5/279.2 |
| PtdEtn 16:0/18:3 | C_39_H_72_NO_8_P | 712.5/255.2 | PlsEtn 16:0/20:4 | C_41_H_74_NO_7_P | 722.5/303.2 |
| PtdEtn 16:0/20:4 | C_41_H_74_NO_8_P | 738.5/255.2 | PlsEtn 16:0/20:5 | C_41_H_72_NO_7_P | 720.5/301.2 |
| PtdEtn 16:0/20:5 | C_41_H_72_NO_8_P | 736.5/255.2 | PlsEtn 16:0/22:6 | C_43_H_74_NO_7_P | 746.5/327.2 |
| PtdEtn 16:0/22:6 | C_43_H_74_NO_8_P | 762.5/255.2 | PlsEtn 18:0/18:0 | C_41_H_82_NO_7_P | 730.5/283.2 |
| PtdEtn 16:0/24:6 | C_45_H_78_NO_8_P | 790.5/255.2 | PlsEtn 18:0/18:1 | C_41_H_80_NO_7_P | 728.5/281.2 |
| PtdEtn 18:0/18:0 | C_41_H_82_NO_8_P | 746.5/283.2 | PlsEtn 18:0/18:2 | C_41_H_78_NO_7_P | 726.5/279.2 |
| PtdEtn 18:0/18:1 | C_41_H_80_NO_8_P | 744.5/283.2 | PlsEtn 18:0/20:4 | C_43_H_78_NO_7_P | 750.6/303.2 |
| PtdEtn 18:0/18:2 | C_41_H_78_NO_8_P | 742.5/283.2 | PlsEtn 18:0/20:5 | C_43_H_76_NO_7_P | 748.5/301.3 |
| PtdEtn 18:0/20:4 | C_43_H_78_NO_8_P | 766.5/283.2 | PlsEtn 18:0/22:6 | C_45_H_78_NO_7_P | 774.5/327.2 |
| PtdEtn 18:0/20:5 | C_43_H_76_NO_8_P | 764.5/283.2 |  |  |  |
| PtdEtn 18:0/22:6 | C_45_H_78_NO_8_P | 790.5/283.2 |  |  |  |
| PtdEtn 18:0/24:6 | C_47_H_82_NO_8_P | 818.5/283.2 |  |  |  |

a) MRM: multiple reaction monitoring
